# 

**Published:** 1915-05-15

**Authors:** 


### CONTENTS.

PAGE

**Military Methods in Medicine** ... ... ... ... 139

**Service and Silence** ... ... ... ... ... ... 140

**Hospital and Institutional News** ... ... ... 141

The Business of War—Wounded Soldiers at Voluntary Hospitals—The Norwich £5 per Bed Scheme—The Monetary Value of Example—Bristol's New War Hospital—Income Tax and Hospital Salaries—A Maternity Centre in the Potteries—Typhus in Serbia—Invalids at the Front—War Changes at the Epileptic Colony — Public Health in Paris — Success of a Woman Pharmacist—Custom and Habit—A Short Way with Defectives—Radium at Manchester Infirmary—Hampstead General Hospital—Aberdeen Doctors and the War—Open-Air Work for Tuberculous Patients—The Work at the Royal Eye Hospital—The Insurance Act in Pembrokeshire—The Shortage of Doctors—Premature Discharges from Ophthalmic Schools—Some Scottish Medical Appointments—The Sinking of the *Lusitania*—A Blot on National Insurance Administration—"Secret Remedies" Again Vindicated—St. Bartholomew's Hospital and Previous Wars—Compulsory Notification of Measles and Whooping Cough—Objections to Partial Compulsion—This Weeks' Drug Market—To Our Readers.

PAGE

**The Early Diagnosis of Pulmonary Tuberculosis** ... ... ... ... ... ... 147

From the View Point of General Practice: By H. Hyslop Thomson, M.D., D.P.H.

**A Medical Causerie** ... ... ... ... ... 149

**The Late Sir William Gowers, M.D., F.R.S.** 150

By B. Burford Rawlings.

**A Mirror of Facts** ... ... ... ... ... ... 151

Some Public Health Reports.

[*See over.*]

### CONTENTS—(*continued*).

| | PAGE |
|---|---|
| **The Speech Clinic at St. Thomas's Hospital** | 152 |
| The Case for General Adoption. | |
| **A Coroner's Report** ... ... ... ... ... ... | 153 |
| Dr. F. J. Waldo's Experiences in 1914. | |
| **Round the World** ... ... ... ... ... | 154 |
| Fellow-Workers and the Roll of Honour. | |
| **The Reserves of Beds in London Hospitals** ... | 155 |
| King Edward's Hospital Fund for London Statistics. | |
| **The Editor's Letter-Box** ... ... ... ... | 156 |
| The Royal Hospital for Incurables. | |
| " A Doctor's Fear of Premature Cremation." | |
| **Personalia** ... ... ... ... ... ... | 156 |
| **Birds in my Garden** ... ... ... ... ... | 157 |
| By a Wounded R.A.M.C. Officer. | |
| **From Pharmacy to Medicine** ... ... ... | 158 |
| **A Hospital for Belgian Soldiers** ... ... ... | 158 |
| **Gifts and Bequests** ... ... ... ... ... | vi |
| **Doctors' Wills** ... ... ... ... ... ... | vi |
| **Medical Appointments** ... ... ... ... | vi |
| **The Book Market** ... ... ... ... ... | vi |
| **The Institutional Worker** ... ... (Suppl.) | 1 |
| VII.—The Out-Patient Clerk. | |
| Question for May. | |
| Questions Invited. | |
| Enquiries and Answers. | |
| The Sick and in Need. | |
| Practical Matters. | |
| Acknowledgments. | |

